# Supplementary material for: Near-Infrared Imaging of Colonic Adenomas In Vivo Using Orthotopic Human Organoids for Early Cancer Detection
Source: Cancers (Basel). 2023 Sep 29;15(19):4795. doi: 10.3390/cancers15194795 (PMC10571995; doi:10.3390/cancers15194795)
Supplement: Supplementary file 1 [file cancers-15-04795-s001.zip › cancers-2613737-supplementary.pdf]

Supplementary Figures

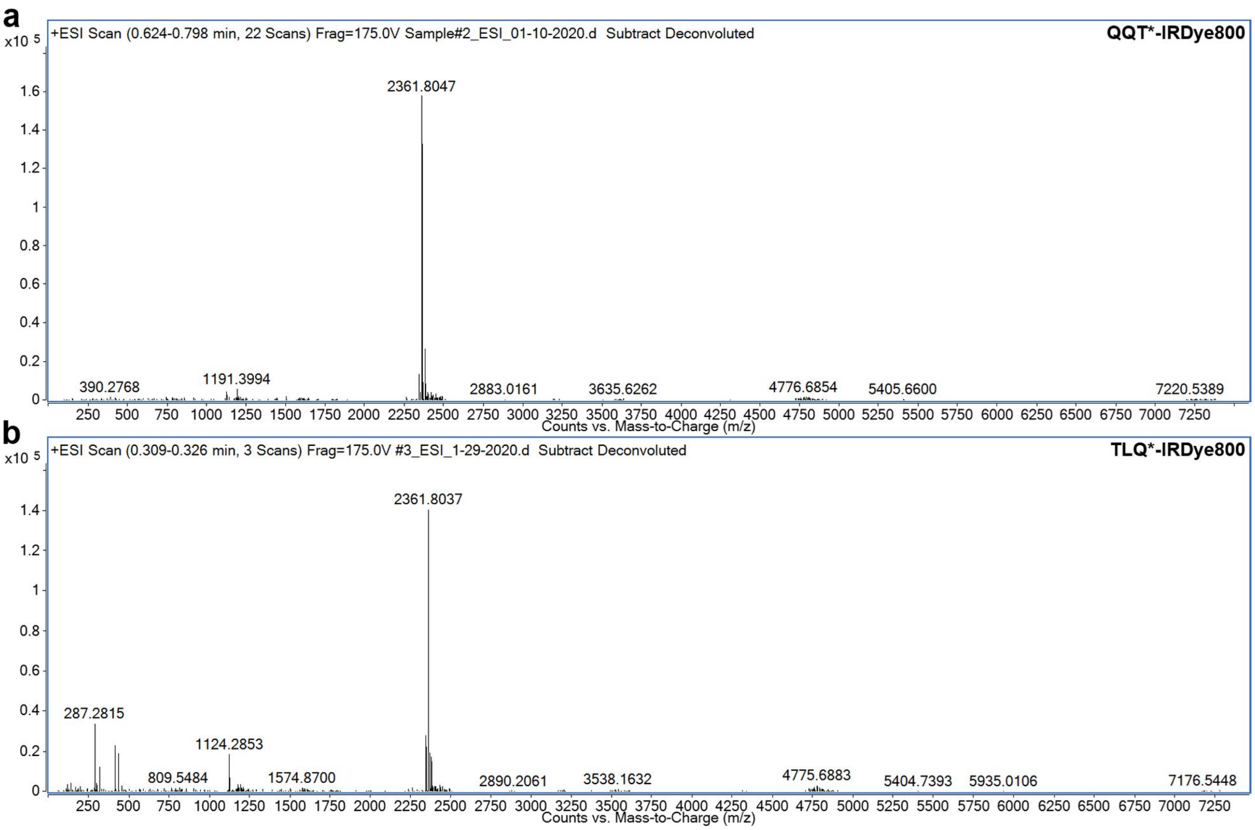

Figure S1 – **Mass spectrometry**. A mass-to-charge (m/z) ratio of 2361.80 was measured for **a**) QQT*-IRDye800 and **b**) TLQ*-IRDye800, which agreed with the expected value of 2361.80 for either peptide.

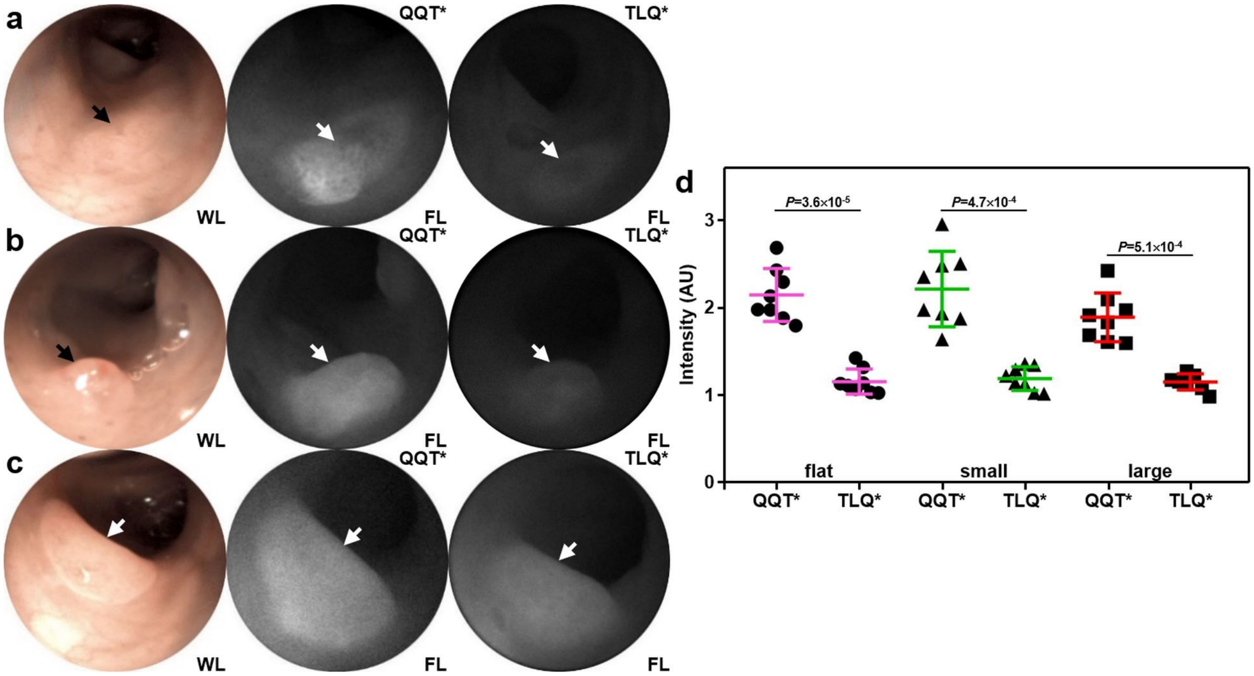

Figure S2 – **In vivo imaging of adenomas with various morphologies**. White light images collected endoscopically show presence of **a**) flat, **b**) small, and **c**) grossly visible spontaneous adenomas in colon of *CPC;Apc* mice. NIR fluorescence images collected 1 hour after intravenous administration of QQT*-IRDye800 show increased intensity from all lesions. Fluorescence images collected using TLQ*-IRDye800 (control) showed minimal intensity from the same lesions. **d**) Quantified fluorescence intensities for QQT*-IRDye800 show significantly higher mean (±SD) T/B ratio from flat lesions, small, and grossly visible adenomas (2.17±0.30, 2.23±0.43, 1.91±0.28) versus TLQ*-IRDye800 (1.17±0.14, 1.20±0.13, and 1.17±0.09). *P*-values were calculated by paired t-test.

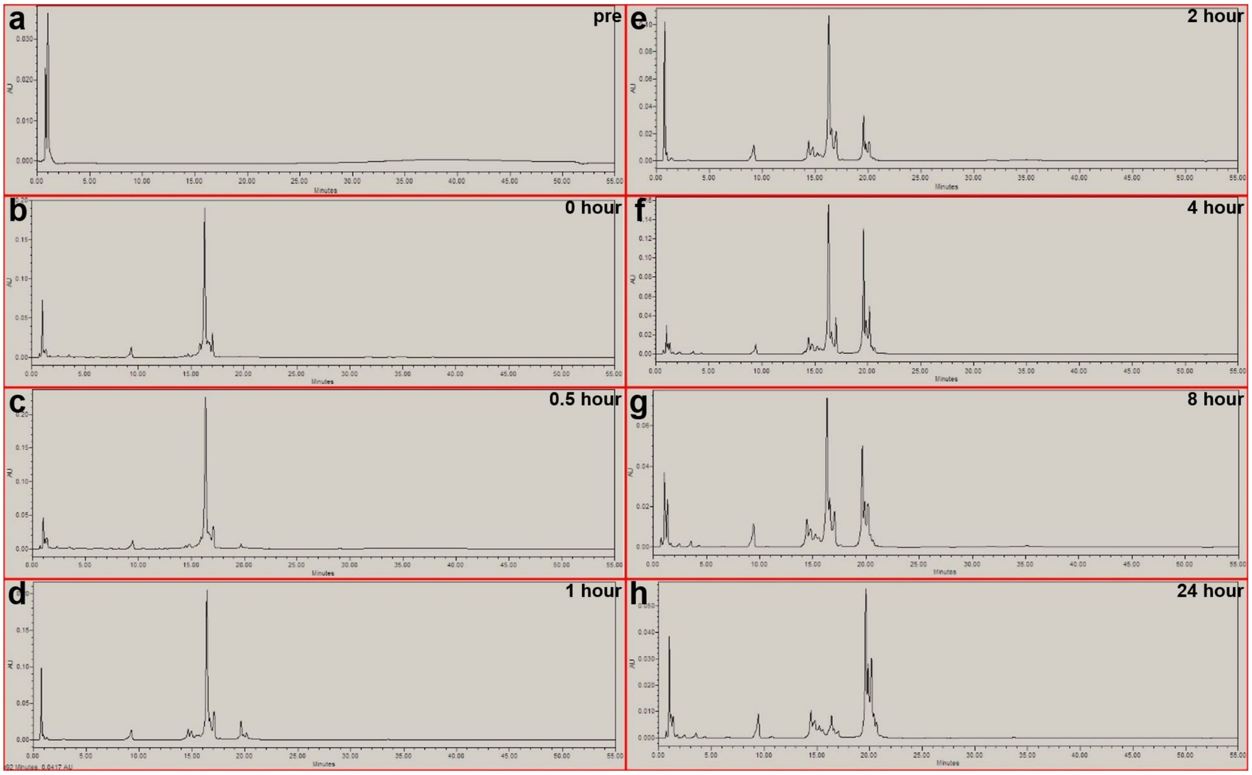

Figure S3 – **Serum stability**. QQT*-IRDye800 at a concentration of 30 μM was incubated in 194 μL of *CPC;Apc* mouse serum for **a**) pre, **b**) 0, **c**) 0.5, **d**) 1.0, **e**) 2, **f**) 4, **g**) 8, and **h**) 24 hours. Serum stability was measured using analytical RP-HPLC. The relative concentration was determined by the area-under-the-peak, and a half-life of $T_{1/2}$ = 3.6 hours was measured, $R^2 = 0.99$.

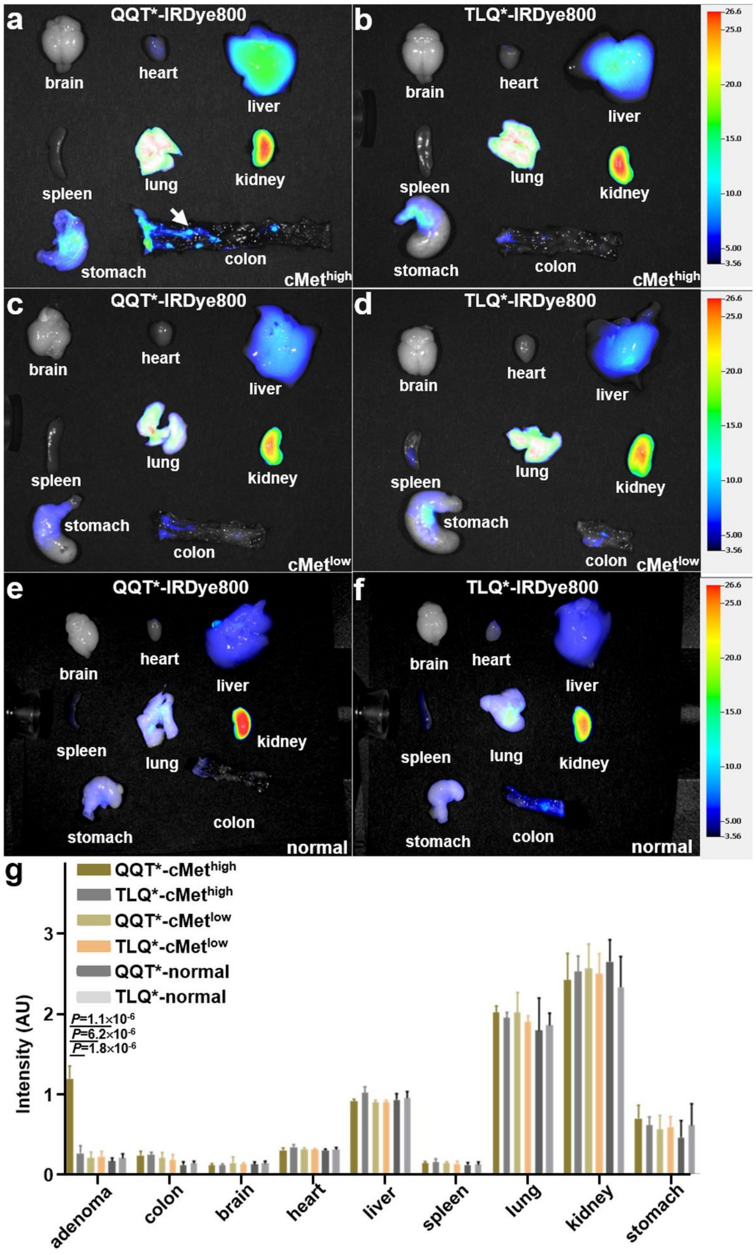

Figure S4 – **Peptide biodistribution**. NIR fluorescence images are shown as an overlay on white light images from major organs at 1 hour post-injection of QQT*-IRDye800 and TLQ*-IRDye800 in NSG mice bearing **a**,**b**) cMet^{high} and **c**,**d**) cMet^{low} adenoma and **e**,**f**) normal human colonoids, respectively, implanted in normal mouse colon. **g**) Quantified results showed uptake of QQT*-IRDye800 by the cMet^{high} adenoma colonoid was significantly higher than that for the cMet^{low} and normal (1.24±0.16 versus 0.23±0.08 and 0.18±0.04). This result was also significantly higher than that for TLQ*-IRDye800 (0.29±0.098, 0.25±0.06, and 0.23±0.6). *P*-values were calculated by unpaired t-test.

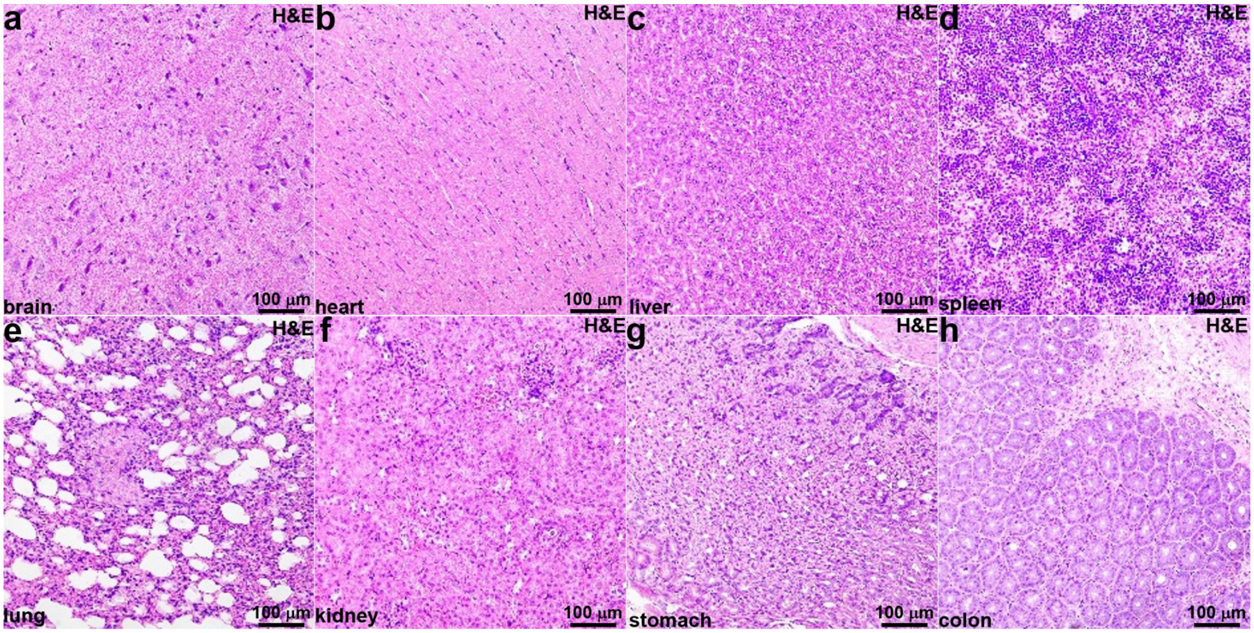

Figure S5 – **Animal necropsy**. NSG mice were sacrificed at 48 hours post-injection with QQT*-IRDye800 (200 μM, 200 μL). No signs of acute toxicity are seen on histology (H&E) of vital organs, including **a**) brain, **b**) heart, **c**) liver, **d**) spleen, **e**) lung, **f**) kidney, **g**) stomach, and **h**) colon.

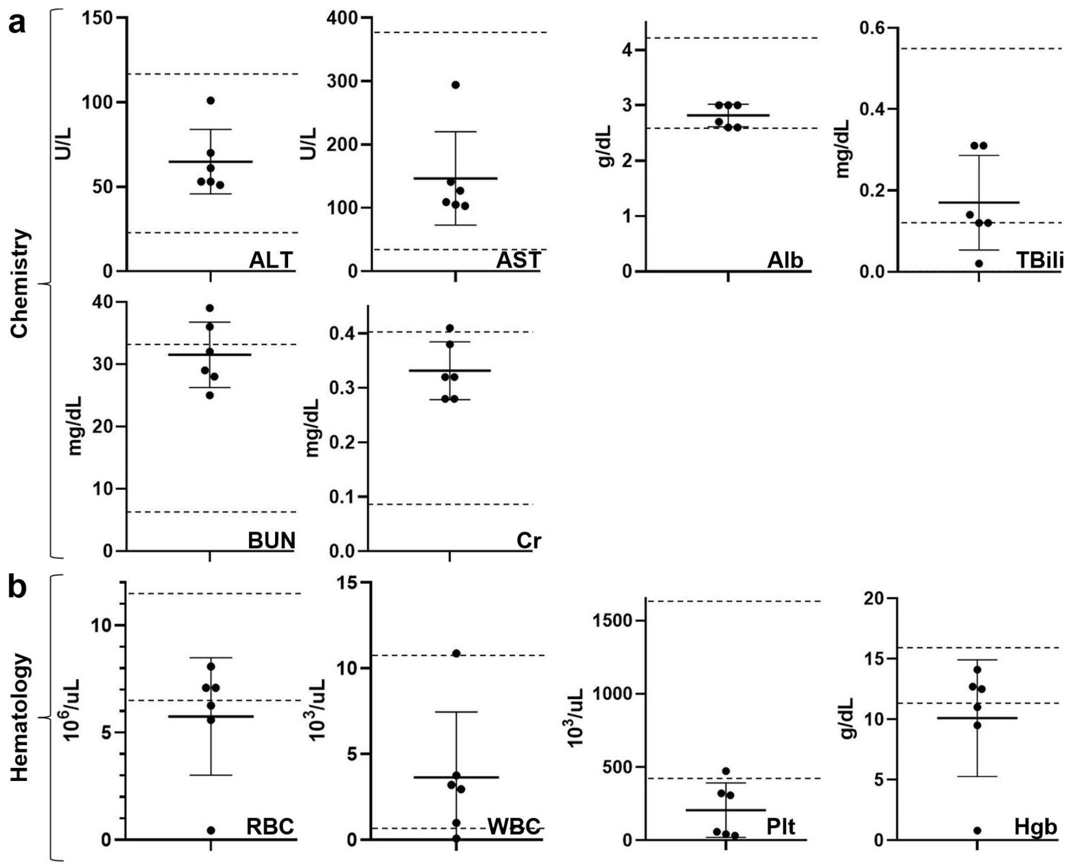

Figure S6 – **Acute toxicity**. Whole blood was obtained from *CPC;Apc* mice at 48 hours post-injection with QQT*-IRDye800 (200 μM, 200 μL). **a**) Results for chemistry, including aspartate aminotransferase (AST), alanine aminotransferase (ALT), albumin (Alb), total bilirubin (TBili), blood urea nitrogen (BUN), and creatinine (Cr), and **b**) hematology, including red blood cells (RBC), white blood cells (WBC), platelets (Plt), and hemoglobin (Hgb), are shown. Mean values were determined from n = 6 mice. Parameters for normal values are identified by dashed lines.
